# *In Silico* Analysis of Cell Cycle Synchronisation Effects in Radiotherapy of Tumour Spheroids

**DOI:** 10.1371/journal.pcbi.1003295

**Published:** 2013-11-14

**Authors:** Harald Kempf, Haralampos Hatzikirou, Marcus Bleicher, Michael Meyer-Hermann

**Affiliations:** 1 Department of Systems Immunology, Helmholtz Centre for Infection Research, Braunschweig, Germany; 2 Frankfurt Institute for Advanced Studies, Frankfurt, Germany; 3 Center for Advancing Electronics Dresden, TU Dresden, Dresden, Germany; 4 Department of Life Sciences, Technische Universität Braunschweig, Braunschweig, Germany; University of Notre Dame, United States of America

## Abstract

Tumour cells show a varying susceptibility to radiation damage as a function of the current cell cycle phase. While this sensitivity is averaged out in an unperturbed tumour due to unsynchronised cell cycle progression, external stimuli such as radiation or drug doses can induce a resynchronisation of the cell cycle and consequently induce a collective development of radiosensitivity in tumours. Although this effect has been regularly described in experiments it is currently not exploited in clinical practice and thus a large potential for optimisation is missed. We present an agent-based model for three-dimensional tumour spheroid growth which has been combined with an irradiation damage and kinetics model. We predict the dynamic response of the overall tumour radiosensitivity to delivered radiation doses and describe corresponding time windows of increased or decreased radiation sensitivity. The degree of cell cycle resynchronisation in response to radiation delivery was identified as a main determinant of the transient periods of low and high radiosensitivity enhancement. A range of selected clinical fractionation schemes is examined and new triggered schedules are tested which aim to maximise the effect of the radiation-induced sensitivity enhancement. We find that the cell cycle resynchronisation can yield a strong increase in therapy effectiveness, if employed correctly. While the individual timing of sensitive periods will depend on the exact cell and radiation types, enhancement is a universal effect which is present in every tumour and accordingly should be the target of experimental investigation. Experimental observables which can be assessed non-invasively and with high spatio-temporal resolution have to be connected to the radiosensitivity enhancement in order to allow for a possible tumour-specific design of highly efficient treatment schedules based on induced cell cycle synchronisation.

## Author Summary

The sensitivity of a cell to a dose of radiation is largely affected by its current position within the cell cycle. While under normal circumstances progression through the cell cycle will be asynchronous in a tumour mass, external influences such as chemo- or radiotherapy can induce a synchronisation. Such a common progression of the inner clock of the cancer cells results in the critical dependence on the effectiveness of any drug or radiation dose on a suitable timing for its administration. We analyse the exact evolution of the radiosensitivity of a sample tumour spheroid in a computer model, which enables us to predict time windows of decreased or increased radiosensitivity. Fractionated radiotherapy schedules can be tailored in order to avoid periods of high resistance and exploit the induced radiosensitivity for an increase in therapy efficiency. We show that the cell cycle effects can drastically alter the outcome of fractionated irradiation schedules in a spheroid cell system. By using the correct observables and continuous monitoring, the cell cycle sensitivity effects have the potential to be integrated into treatment planing of the future and thus to be employed for a better outcome in clinical cancer therapies.

## Introduction

Tumours are complex dynamic objects which can adapt to changes in their environmental conditions and accordingly react to treatments such as radiotherapy. Withers was one of the first to note that the now common scheduling of radiotherapy in fractions is efficient, because it exploits these dynamic intra-tumoural effects. He identified and described the four “R”s of radiotherapy which today form the basis of clinical practice: redistribution, re-oxygenation, repair and regrowth. After the use of fractionation schemes became common in clinical treatment, further investigation led to the conclusion that standardised protocols might not be the optimal solution for each patient, but rather that altered individual fractionation schemes should be considered [1]. In particular the cell cycle redistribution during radiotherapy has been studied early [2], [3] and regularly ever since in a variety of experimental systems [4]. Nevertheless, today cell cycle effects are not routinely included in treatment planning and are disregarded as “unusable” even though the advent of modern imaging technologies has delivered a variety of suitable tools which could assess not only oxygenation but also cell cycle status *in vivo* [5], [6].

Cancer therapy is clearly advancing in the direction of highly individualised, tailored treatment protocols as a result of a range of new technological developments in radiation delivery [7] and monitoring [8], [9]. In order to find optimal protocols, a detailed understanding of the treatment effects on the target system is necessary. This is where mathematical and computational models are needed in order to describe and understand the complex interdependencies of the tumour. They open up the possibility to also screen unusual treatment approaches for efficient strategies. Accordingly, over the last decade, a variety of models have been designed for the purpose of treatment planning, be it for radiotherapy [10], [11], chemotherapy [12], combined treatment approaches or others aspects of tumour growth and therapy [13]–[15].

One particularly successful example of therapy optimisation is the description and use of circadian timings in cancer therapy [16], [17]. Especially for chemotherapy the careful timing of drug delivery in conjunction with the natural cell cycle dynamics has led to interesting predictions [15], [18], [19] and an measurable increase in clinical efficiency both in cancer-therapy and in the treatment of non-cancer diseases [20]–[22]. Also with respect to DNA repair and gene expression, circadian cell cycle timings are of interest for cancer therapy [23]. However few models have specifically addressed the effect of cell cycle redistribution in conjunction with cell-cycle specific radiosensitivity [24] and most of these rely on an abstract representation of the tumour cell population. In comparison to a previous single cell-based model by the authors [25] the new model relies exclusively on measurable cell parameters in order to allow for a more direct comparison to experiments. It has been based on the linear-quadratic model for radiation survival and introduces a range of observables to quantitatively describe the synchronisation and sensitivity changes within the tumour spheroid. These qualitative changes and extensions were necessary in order to allow for the study of realistic fractionation schemes as well as alternative radiation delivery timings. Tumour spheroids have been chosen as model system for radiation reactions as they allow for a straightforward testing of predictions *in vitro*, while retaining a considerable degree of realism when compared to flask cultures [26]. It is to be expected that the effects of synchronisation observed in tumour spheroids are not completely lost in *in vivo* tumours and are worth being a target of further research for that reason.

Within the investigation the focus rests on the redistribution of cells within the cycle phases which occurs as a result of irradiation during treatment. Using a three-dimensional, agent-based model of microtumour growth, we will show its implications for the fractionation of irradiation during clinical treatment schedules. It allows us to demonstrate that an individualised treatment plan, which incorporates cell cycle redistribution effects, can yield a better outcome than typical standardised treatment schedules. The predictions of our model system can thus be used as a guideline for subsequent *in vitro* experiments and, after *in vivo* study and validation, ultimately be incorporated into clinical trial settings.

## Methods

### Agent-based tumour spheroid growth

A three-dimensional single-cell based model is developed in order to study the growth of tumour nodules and their reaction to therapeutic approaches. The main parameters are listed in table 1. It has to be stressed that all parameters used within the simulation are physically accessible and thus can be obtained from experimental measurements. Accordingly the simulation can be tailored to model a specific cell line in conjunction with joint experimental investigations. However the observed effects are of a universal nature, meaning that they are largely insensitive to variation of parameters, as has been tested in the simulation. Hence the choice of parameters is exemplary for a wide physiological range of cells and does not aim to reflect one specific cell line. Technically the present model is developed in C++ code on the framework of the Voronoi-tessellation of biological tissue [27], [28]. A validation of the employed tumour growth model is provided in reference [25] and in the supporting figure S3.

**Table 1:** Selection of parameters for growth and irradiation used within the simulation.

Parameter	Value	Remark
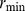 / 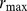	7.94/10 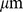	minimum and maximum cell radius [86]
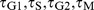	8 h, 6 h, 4.5 h, 1 h	average cell cycle phase duration [43], [87]
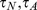	24 h, 12 h	average necrosis and apoptosis duration [40], [51], [87]
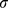	0.3	standard deviation of phaselength normal distribution, fit
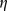	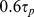	cut-off for phaselength normal distribution, fit
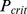	200 Pa	critical cell pressure for quiescence; [88], growth fit
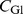	0 mM	glucose concentration for necrosis; growth fit
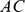	0.66	chance for acute, fast radiation-induced cell death; [57] fit
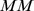	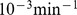	effective rate of slow cell death at G2/M checkpoint; [57] fit
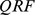	1.5	dose-reduction factor of quiescent cells [47], [49]
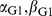	0.351, 0.04	LQ-parameters for G1 cells (and G0 with QRF) [2] [46]
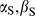	0.1235, 0.0285	LQ-parameters for S-phase [2] [46]
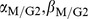	0.793, 0	LQ-parameters for M- and G2-phase [2] [46]

Further parameters and sources for the handling of cell interaction, nutrient diffusion and consumption can be found in [25] and [28]. The model aims to describe a generic tumour so the analysis does not rely on data for one specific tumour cell line only but on parameters which are within the established physiological range. Parameters for glucose and oxygen diffusion from [89] [90] [91] and according cell uptake rates from [44], [86].

The use of a three-dimensional spheroid model is of importance in order to obtain a system which comprises a range of features that are present in real tissues and which cannot be adequately described using two-dimensional models [29], [30]. Accordingly it has been demonstrated experimentally that the treatment reaction of cells in three dimensional structures such as multilayers, spheroids or xenograft tumours can differ strongly from the reaction in a monolayer [31]–[34]. This is to a large extent an effect of the cell interaction within a tissue and the specific spatially and temporally heterogeneous cell cycle distribution which will arise in a tumour spheroid [35], [36]. Realistic nutrient gradients, as they develop in response to diffusion through a breathing tissue, will only be found in such three dimensional cell arrangements.

Overall a macroscopic tumour *in vivo* (with a diameter in the order of centimetre) is comprised of small microscopic sub-volumes of about 500 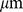 diameter which form in between capillaries. Each of these microtumour regions will consist of an outer proliferating rim, an intermediate mostly quiescent region and an inner necrotic region as a result of the limited nutrient diffusion range. Due to the structure of vessels these regions will usually be elongated and stretch out between capillaries but also regular patterns of nutrient support have been observed in tumours [37]. Our model spheroid directly corresponds to one such microregion or tumour nodule [36], and can also serve as a model for the reaction of a larger tumour volume as a result of its functional and histological correspondence to a microtumour region [38].

#### Cell representation and cycle

The spatial arrangement of cells in a tissue is represented using a Voronoi-Delaunay approach [27], [28]. Interaction between cells is adhesive-repulsive and performed using the Johnson-Kendal-Roberts model [39] as described in detail in [25].

**Figure 1:**
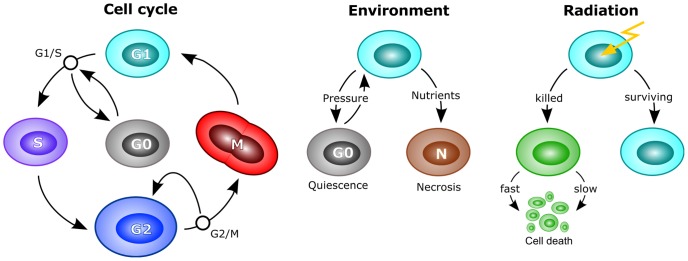
Cell cycle, response to environmental factors and radiation as implemented in the model. Black circles mark cell cycle checkpoints. Cells can enter and leave quiescence in response to the local pressure at the G1/S checkpoint. If the critical conditions improve, cells re-enter the active cell cycle by passing the restriction point. Growing cells double their volume during G1 and G2 phase, so that the cell volume is conserved in mitosis. At the G2/M checkpoint cells will be halted if their DNA is damaged. This arrest is subject to a chance of failure, so that, with a defined probability, cells can pass into mitosis even though their DNA is damaged. Cell death in response to critical nutrient deprivation is possible at any time via necrosis. In response to irradiation, individual cells will commit to cell death if a random value exceeds their cell-cycle specific survival chance from the linear-quadratic model in equation 1. Cell death in response to radiation is realised either via a fast, acute commitment to cell death or by prolonged fixation at the G2/M checkpoint which will lead to cell death via apoptosis as a result of mitotic catastrophe or other fatal errors.

Cell cycle progression is assumed to depend on external conditions, specifically the local nutrient availability (glucose concentration in the medium) and interaction with neighbouring cells (integral pressure) as shown in figure 1. Within the model a complete local depletion of glucose will trigger cell death via a fast necrotic process [40], while an integral pressure on the cell above 200 Pa will induce quiescence at the G1/S-checkpoint as a result of contact inhibition [35], [41], which lasts until the pressure falls below the threshold value.

The spheroids used for irradiation within the scope of this investigation are grown from a small number of 10 virtual seeder cells which resemble cells of the EMT6 line in *in vitro* cultures [42] (matching the typical cell cycle phase-lengths, interaction parameters, response to starvation and so forth, see table 1 and [25]).

#### Nutrient modelling

Availability of glucose and oxygen is modelled using a cubic reaction diffusion solver system of 1.4 mm edge length. Nutrient conditions in regions of the system which are not occupied by cells are adapted to match *in vitro* values from [43] for growth verification as described in [25] or to typical tissue concentrations with 5 mM glucose and 0.13 mM oxygen when *in vivo* tumour nodule growth is studied. Cells consume nutrients using a cell cycle phase-specific uptake rate and accordingly act as sinks in the reaction diffusion solver [44], [45].

### Model for cell reactions to irradiation

The total amount of cell death in response to a radiation dose matches experimental measurements, as the linear quadratic model for single cell survival with measured parameters is employed. In response to irradiation with the dose *D* (defined in Gy) cells obtain a *cell cycle phase-dependent* survival probability 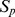 from the linear quadratic model [46]:
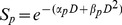
(1)As physiological example 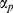 and 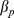 values of V79 hamster cells which were subjected to x-rays by Sinclair [2] are employed (supporting figure S2 and table 1). It has been repeatedly reported that quiescent cells exhibit an increased resistance to radiation damage [47]–[50]. This fact is incorporated into the model by using a quiescence resistance factor (QRF = 1.5) to scale down the effective radiation dose which quiescent cells experience. Thus, within this assumption, quiescent cells use the measured LQ-parameters of G1 cells but with reduced dose.

Once committed to the death path, a cell can either be killed on a fast timescale (probability “acute chance” 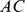) or after delay on a slow timescale (with probability 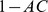) as shown in figure 1. The fast process corresponds to a relatively acute, direct commitment to cell death via apoptosis or necrosis in response to heavy DNA damage (e.g. clustered lesions) and accordingly a rather low duration for cell death was chosen with an average of 12 h [51]–[53]. The slow process corresponds to a prolonged inability to pass the G2/M checkpoint which will lead to the pile-up of cells in the G2-phase after irradiation and eventually leads to cell death e.g. via mitotic catastrophe or a loss in the so called “race between repair and cell death” [54], [55]. Both is represented as failure at the G2/M checkpoint and progression to cell death with a “mitotic mismatch”-rate 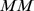.

While this model drastically simplifies the multitude of mechanisms of radiation-induced cell death [56], the overall amount of cell death observed will be in agreement with experimental measurements within the LQ-model. The inclusion of a fast and slow damage timescale increases the matching of the predicted cell cycle response to experimental measurements [57]. Damage repair is not considered in detail within the model as it will be phenomenologically contained within the measured LQ-survival. Furthermore the typical radiation delivery interval within the simulations will be large enough in order to assume largely independent irradiation events as the majority of remaining damage will have been repaired in the inter-fraction time [58], [59].

### Measuring radiosensitivity and tumour burden

In order to assess the radiosensitivity of the tumour spheroid, we use the ratio of the virtual total survival observed in our simulation at the time of interest and a baseline survival which is expected for the tumour cells under consideration. The expected survival 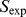 is defined as the average of the survival probabilities, where each cell cycle phase specific survival probability from the LQ-model 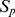 is weighted with the average duration of the corresponding phase-length 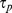 and normalised using the total average cycle time 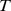:
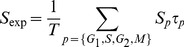
(2)This baseline survival reflects the typical survival of an exponentially growing tumour spheroid without quiescent sub-population and with uniform distribution of the cells proportional to the cycle phase-lengths. Consequently it should correspond to the expected survival within fully active microregions of a macroscopic tumour. However, within the scope of this work it will be only applied in the context of tumour spheroids.

The observed cell survival 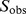 can be obtained at any time by virtual simulation of the impact of a dose of radiation, without application of the according changes to the tumour system. The fraction of surviving cells yields the observed survival:
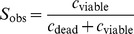
(3)Consequently we define the enhancement 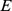 as the ratio of expected and observed survival:
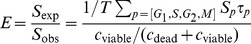
(4)An enhancement larger than one reflects a tumour in a state of increased sensitivity to radiation, while a lower enhancement reflects a resistant state, as is the case for a tumour which contains a large quiescent population.

As a measure of treatment success we use the tumour burden, which is defined as the integral of the total number of cells in the tumour over a time of interest (area under the curve). A typical unit for this observable is 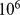 cell-days. Further radiobiological observables like the mitotic index (MI) and S-phase fraction (SPF) are directly accessible from the cell cycle distribution of the agent-based model at all times. They can be used to predict radiosensitivity directly as in [60] and can be compared to experimental measurements.

### Measurement of cell phase-angles and tumour synchronicity

The cell phase-angle 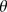 is used to measure the relative progression of an individual cell through its cell cycle, independent of functional cell cycle phases. 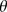 is defined as the ratio of total time spent in the active cell cycle phases 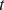 (cells which enter quiescence will thus not advance their phase-angle) and the individual total cell cycle time 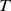:
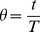
(5)Since the cell cycle times are drawn from a normal distribution (with a maximum variation) individually for every cell and cycle phase, two cells can have an identical phase-angle 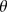 while their functional cell cycle phase is not identical.

Using the phase-angle we define the orderedness 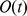 of the tumour cell population, by calculation of the Shannon entropy of the system. The probability mass function 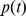 will be obtained by sorting all cells of the tumour into 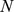 bins according to their cell phase-angle 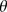. Thus we can calculate the Shannon entropy of the tumour system
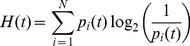
(6)and use its maximum 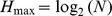 to define the orderedness of the population as
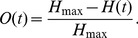
(7)The entropy and orderedness of the system are well behaved, so that it is possible to use a small number of bins 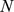 for grouping. One such arrangement is the ordering of cells by *functional* cell cycle phase or cell DNA content, which are both easily assessed experimentally in *in vitro* settings or *in vivo* from biopsies.

The orderedness 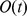 of the system will approach 1 for synchronous populations and 0 for populations which are uniformly distributed in the cell cycle.

## Results

### Growth and cycle desynchronisation

*In silico* tumour spheroids were grown in a standardised protocol from 10 tumour seeder cells using the parameters in table 1. The seeder cells were allowed to grow for 14 days and formed microtumours of about 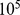 cells with a typical diameter of 700 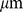. An initial exponential growth phase was followed by a subsequent growth retardation by induced quiescence and necrosis. Treatment of the spheroids started at day 14.

**Figure 2:**
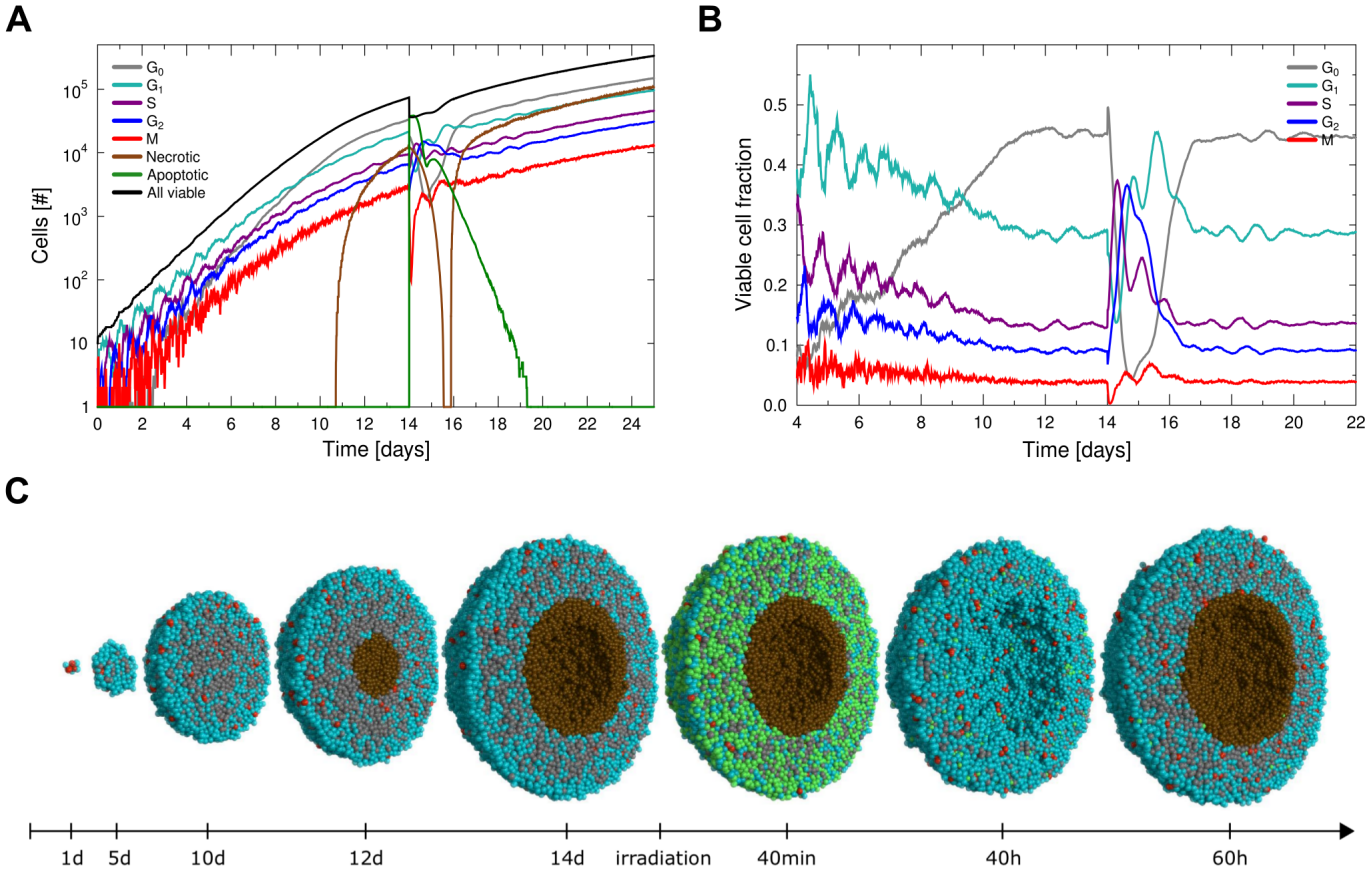
Reaction of a tumour spheroid to irradiation. Panel **A** shows the cell phase distribution during growth of a tumour spheroid and in response to irradiation with 4 Gy. **B** Redistribution in response to 4 Gy and subsequent dynamics in the fraction of viable cells. **C** Lateral cut through a tumour spheroid during different phases of growth and irradiation. An initial small number of seeder cells will form a solid tumour spheroid, where cells in high-density regions go into quiescence. Nutrient deprivation and subsequent dissolution of necrotic cells lead to the formation of a hollow core. After irradiation with 4 Gy a majority of cells will be apoptotic, which leads to a reactivation of quiescent cells. Consequently a fast regrowth and the re-establishment of the necrotic core are observed. Cells are visualised as spheres but are handled as polyhedra while in contact within the 3D Delaunay triangulation used in the model [25]. Cells in G1, S or G2-phase in cyan, mitotic cells in red, quiescent cells in grey, necrotic cells in brown and apoptotic cells in green.

The fully grown microtumours incorporated all typical histological regions which are of importance for the radiation response. A large, stable quiescent cell population was present, which could quickly respond to radiation-induced changes in the tumour environment (figure 2). Due to dissolution of necrotic cells a hollow core formed in the tumour spheroid before a treatment plan was started (figure 2 and supporting figure S3

The synchronicity of the tumour cell population steadily decreased over time as the cell cycle progression was desynchronised by the normal distribution of cell cycle times. This decrease is visible as smoothing of the oscillation in the cell cycle distribution in figure 2A and directly via the decrease in orderedness as shown in supporting figure S1. Another major contribution to the desynchronisation was the entry of cells into quiescence and subsequent re-entry into the active cycle.

### Irradiation reaction, cycle redistribution and enhancement

After homogeneous irradiation of the tumour spheroid with 4 Gy a large fraction of cells committed to cell death (figure 2). However, irradiation of the tumour also led to its subsequent reactivation. Through the clearing of dead cells the pressure and nutrient situation for surviving cells improved considerably, which triggered a fast re-entry of previously quiescent cells into the active cycle (figure 2), as has been observed experimentally [61], [62]. This radiation-induced regrowth was exponential as almost all clonogenic cells in the spheroid were dividing again.

**Figure 3:**
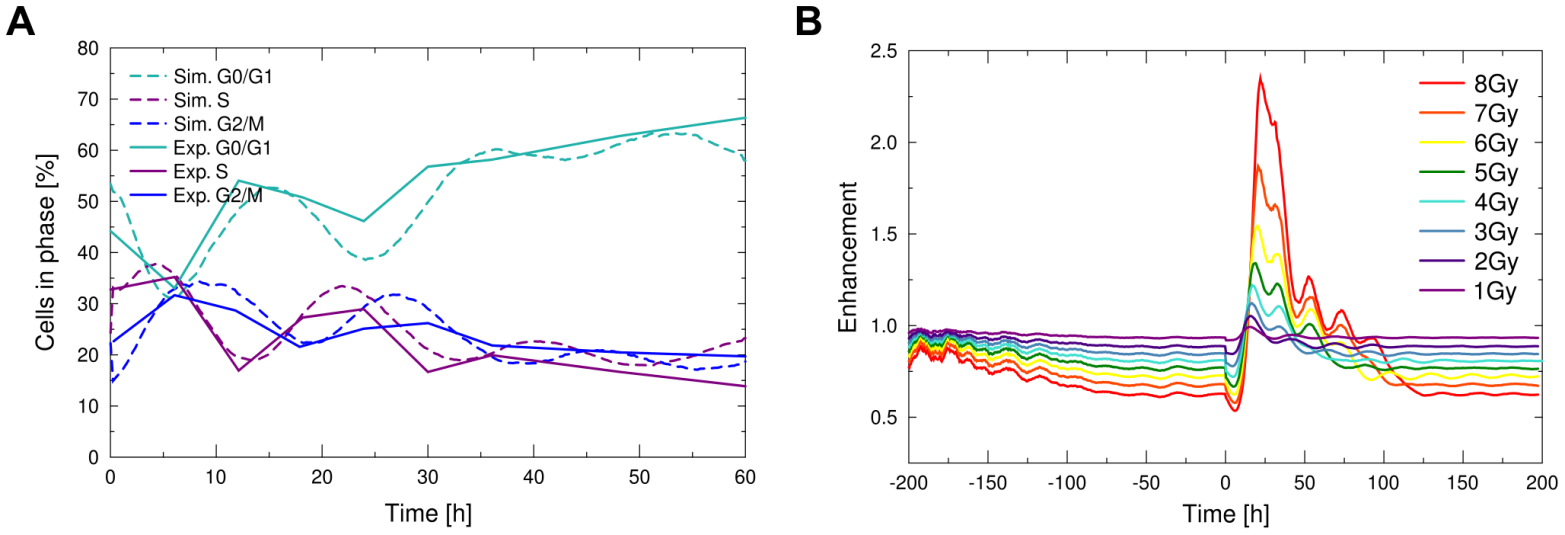
Cell phase redistribution and according change in overall radiosensitivity in response to irradiation. A Comparison of the cell cycle redistribution *in silico* after irradiation with 2 Gy and *in vitro* for LN229 cells from [57]. **B** Effect of a single radiation dose on post-irradiation sensitivity of the tumour. Depending on the applied dose the effects of a growing quiescent, radioresistant sub-population are increasing, as can be seen in the development of the enhancement during the tumour growth up to the irradiation at time zero. After irradiation an initial period of increased radioresistance is followed by transient maxima in radiosensitivity which are suitable for targeting by subsequent fractions. Oscillations of enhancement are dampened by the entry of cells into quiescence after regrowth and by the normal distribution of cell cycle phaselengths.

Radiation led to a redistribution and synchronisation of the cell cycle progression as it killed predominantly cells in sensitive phases of the cycle. The observed redistribution and subsequent evolution of the cell cycle distribution corresponded well to experimental observations [57] (figure 3). A G2-block of cell cycle progression was observed, where DNA damaged cells gathered at the G2/M checkpoint. Thus the ratio of cells in G1 to cells in G2 was transiently inverted in response to a radiation dose (figure 2). Best agreement was achieved when a high degree of fast, acute and a lower level of slow cell death e.g. through mitotic catastrophe were used for the radiation death dynamics. The timescale but not the quality of the dynamic reaction is subject to variations by cell- and radiation type as can be seen in [58] for Chinese hamster V79 lung cells or [63] for SiHa xenograft tumours.

Due to the higher radioresistance of quiescent cells, immediately after irradiation the relative fraction of quiescent cells among all viable was temporarily increased. The subsequent re-entry of quiescent cells into the active cycle was largely synchronised at the G1/S checkpoint (figure 2).

The synchronisation of the cell cycle progression led to collective oscillations of radiosensitivity in the tumour (figure 3). The enhancement in the tumour exhibits a transient, two-peaked reaction to irradiation. The observed loss of sensitivity for a quiescent tumour and the subsequent gain in sensitivity after irradiation increased with dose. While a quiescent tumour was only half as sensitive to a dose of 8 Gy as its exponentially growing counterpart, after irradiation its sensitivity increased more than twofold. Accordingly, one goal in experimental scheduling can be to design a radiation delivery which is optimised to use these recurring periods of transient sensitivity and avoid dose delivery during times of radiation resistance.

### Comparison of clinical irradiation protocols

Clinically a large integral dose will be applied in multiple fractions in order to sterilize a tumour or reduce its size. Dose delivery will be fractionated in order to limit side effects in surrounding tissue and exploit the initially mentioned effects that the fractionated delivery has on the tumour [1]. The timing of dose application is typically chosen such as to provide a balance between practical restrictions such as clinical workload, curative effect and side effects.

The standard clinical radiotherapy protocol is the repeated application of doses of 2 Gy each in daily fractions which will be administered over a prolonged time until an integral dose of typically 60 Gy is reached. Treatment is often paused during weekends to allow for tissue regeneration and re-oxygenation, but also for reasons of clinical workload. Common alternative fractionation schedules include hyperfractionation e.g. with the delivery of 2 smaller fractions every 12 hours or hypofractionation with the delivery of higher single doses and a shorter total treatment time [46], [64], [65]. Typically a similar integral dose is used (table 2). Alternative schedules which employ very high single doses as in Stereotactic Body Radiation Therapy [66] or oligofractionation [67] will no be part of the investigation, as they would most likely exceed the validity of the linear-quadratic model.

**Table 2:** Overview of selected clinical fractionation schemes that have been tested in the simulation.

Schedule	Total dose	Dose/Fraction	Fractions
	[Gy]	[Gy]	per day/per week
Conventional	60	2	1/5
Accelerated Conv.	60	2	1/7
Hypofractionated	60	4	1/5
Accelerated Hypo.	60	4	1/7
Hyperfractionated	60	1	2/10
Accelerated Hyper.	60	1	2/14
CHART	54	1.5	3/21
Split course	60	2	1/5
Concomittant boost	60	2	1–2/5–10

**Figure 4:**
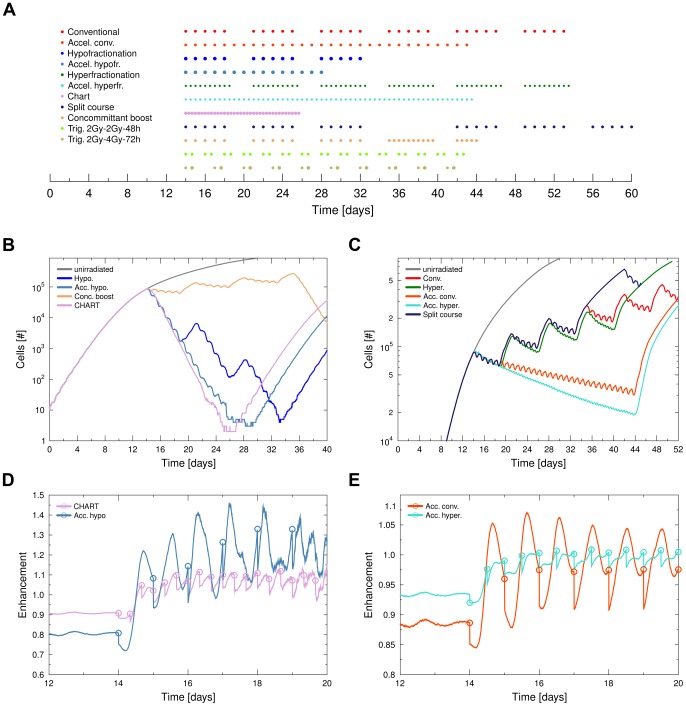
Effect of different treatment schedules on the tumour spheroid. **A** Visualisation of the radiation timing in selected fractionation schedules is provided in table 2. Marker size is indicative of fraction dose. At time zero the tumour is seeded with a small number of cells. Treatment schedules were started at day 14 of tumour growth, when a fully structured tumour spheroid had developed. **B–C** Comparison of total tumour size during high dose-per-time and low dose-per-time scheduling (left >2 Gy/24 h, right ≤2 Gy/24 h ). **D–E** Development of enhancement during selected schedules can explain the different performance of the schedules (radiation times marked with circles).

For better comparison of effects the integral dose for all runs has been chosen to be 60 Gy (except for CHART treatment with 54 Gy). In order to test the results of hypo- and hyperfractionation, extreme cases with doubled or halved doses per fraction where chosen. A visualisation of the according fractionation timings is presented in figure 4.

Figure 4 provides an overview of the effects of selected fractionation schemes from table 2 when applied to the model tumour. In general a high degree of regrowth in response to irradiation was observed *in silico*. Reactivated cells repopulated the tumour and due to their unlimited replicative potential lead to a quick reformation of the spheroid. This was true even when only a very small number of cells was left alive. A typical integral dose of 60 Gy thus did not fully sterilize the model tumour, even when applied in a short amount of time such as in a hypofractionated schedule. This is in agreement with experimental observations on multicellular tumour spheroids *in vitro*, where a much more rapid growth of spheroid cells is observed than for cells in an *in vivo* setting [58].

In terms of a reduction of the tumour burden, the high dose-per-time schedules all performed better. In general they allowed less regrowth of the tumour to occur due to the shortened treatment time. Furthermore they benefited from the quadratic term in the dose-survival relation of the LQ-model eq. 1 due to the high single-doses used.

Longer treatment pauses, as in the conventional, “un-accelerated” schedules, had a significant negative effect on the tumour control. Each pause allowed for an unchecked period of regrowth within the tumour, which was not cancelled out, as the integral dose was kept constant. Treatment pauses can make all the difference between the achievement of a steady reduction in tumour load, or a failure to keep the tumour in check (figure 4).

Schedules which employed a low dose per fraction (such as hyperfractionation) performed better than schedules which delivered the same dose per time in medium-sized single fractions. This is not to be expected, as the quadratic survival term in the LQ-model will yield a lower survival for larger doses. The reason for this observation is the timing of the radiation delivery in conjunction with the development of tumour radiosensitivity (figure 4). While the conventional radiation schedule delivered follow-up doses at a time of low tumour radiosensitivity, within the hyperfractionated schedule follow-up doses were delivered at a time of high radiosensitivity.

Dose delivery within the conventional, accelerated conventional or split course treatment occurred in intervals, which failed to induce a persistent high enhancement in the tumour. Hyperfractionated schedules in contrast succeeded at keeping the enhancement in the tumour at a steady high level, which was especially true for the accelerated hyperfractionation schedule. Effectively the hyperfractionated schedule suppressed the reformation of a radioresistant quiescent subpopulation. Although it allowed the tumour to grow exponentially at all times, the frequent delivery of doses kept the growth in check.

Even so CHART used lower single doses it was able to achieve a high tumour control at an overall lower integral dose. However, the dose per time interval which is applied in CHART treatment is very high with 4.5 Gy/24 h, thus possibly increasing side effects of the treatment. Considering the fast repair of sublethal damage in most cells, CHART would however allow for repair of most damage in surrounding tissue with a delivery interval of 8 hours. CHART-fractionation kept the enhancement of the tumour for follow-up doses steadily above a level of one, thus achieving a moderate increase in effectivity (figure 4).

### Systematic variation of dose rate

**Figure 5:**
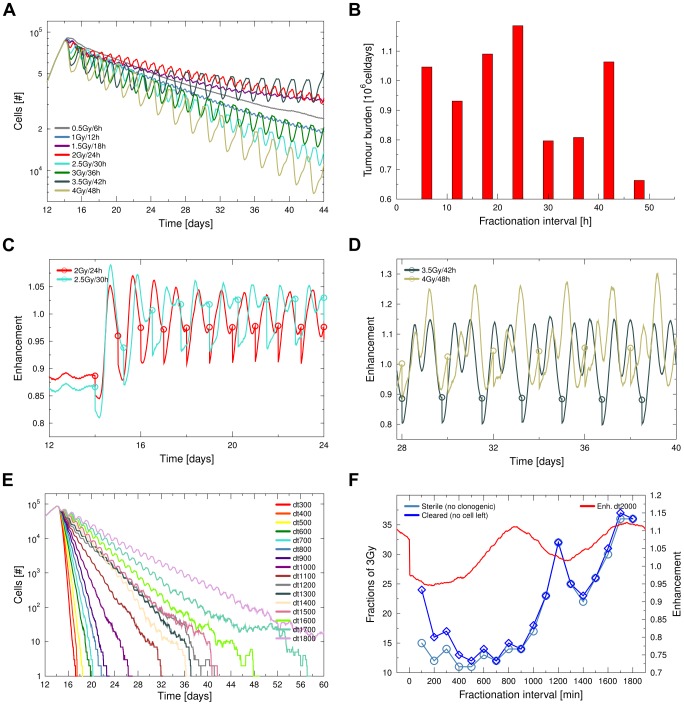
Systematic investigation of the performance of different scheduling schemes. **A** Comparison of total tumour size in response to altered scheduling of the standard dose of 2 Gy/24 h. While high-dose fractions have an advantage because of the quadratic term in the LQ radiation response, they are outperformed by some lower-dose schemes due to a better timing of the treatment to the tumour radiosensitivity development (compare e.g. 2 Gy/24 h and 2.5 Gy/30 h). **B** Overall performance of varied dose distribution measured as tumour burden for the time period from treatment begin at day 14 to day 44. **C–D** Timing of fractions in relation to the enhancement development for selected runs can explain the different schedule performance. **E–F** Repeated delivery of doses of 3 Gy with different delivery intervals until full sterilisation is achieved. The number of fractions required for sterilisation depends non-linearly on the inter-fraction time. This complex dependency is a result of the enhancement development within the tumour as discussed in the text and further illustrated in figure S6.

For a better comparison of the effects of delivery timing, it is useful to systematically compare schedules which apply the same integral dose over the same time, but with a systematically varied dose per time interval. We thus investigated how the varied fractionation of a typical constant dose per time of 2 Gy per day would influence the outcome of a radiotherapy regimen (figure 5). The tumour burden was significantly different and best performance was observed for delivery intervals of 30, 36 and 48 hours (figure 5).

Larger single fractions, as for a delivery interval of 48 h, have the advantage of inducing a higher amount of cell death when compared to the combination of multiple smaller doses (due to the quadratic term in the LQ model). While it is thus not surprising that a run with the largest single doses of 4 Gy showed a good performance, it is interesting that this performance was closely matched by a run with single doses of only 2.5 Gy. Treatment with intermediate single doses of 3.5 Gy performed significantly worse than with doses of 2.5 Gy, which demonstrates that the quadratic dose-effect alone does not determine the success of the treatment. Instead the success of the 2.5 Gy schedule can be explained by the good match between the fractionation timing an the tumour enhancement development (figure 5). A negative timing effect is present in the 3.5 Gy schedule when compared to the 4 Gy schedule (figure 5). The enhancement effects cancel out the advantage of the larger single dose due to LQ-survival.

### Tumour sterilisation

Repeated delivery of doses of 3 Gy with varying inter-fraction time were applied until the *in silico* tumour was fully sterilised (figure 5). Due to the radiation-induced reactivation and regrowth, longer inter-fraction times will be associated with a higher amount of tumour regrowth, so that a linear dependency of total dose necessary for sterilisation and fractionation interval might be expected, which turns out to be wrong. Instead the required number of fractions for sterilisation exhibits a minimum at fractionation intervals of 500–700 minutes.

Analysing the development of enhancement during the continued radiation delivery reveals that the nature of the fractionation curve can be explained by the relation between irradiation interval and enhancement development (see also supporting figure S6). Low fractionation interval times of 100 to 300 minutes are inefficient, because the tumour is still in a region of low enhancement when it receives a follow-up dose. A follow-up interval of 400 minutes already allows for a gain in enhancement before the next dose is applied. This gain in enhancement is so large that it counterbalances the effect of tumour regrowth for treatment intervals from 400 to 1000 minutes. If a larger interval is used, the number of fractions needed to sterilise the tumour grows drastically as the follow-up irradiation coincides with a minimum in enhancement at the 1200 minutes interval.

For even larger fractionation intervals a lower integral dose will be sufficient for sterilisation even though a higher total regrowth time is allowed. The coincidence of rising triggered enhancement and follow-up radiation dose delivery leads to the local minimum in fractions needed between 1300 and 1600 minutes fractionation interval time.

### Triggered and automatic enhancement-based irradiation protocols

**Figure 6:**
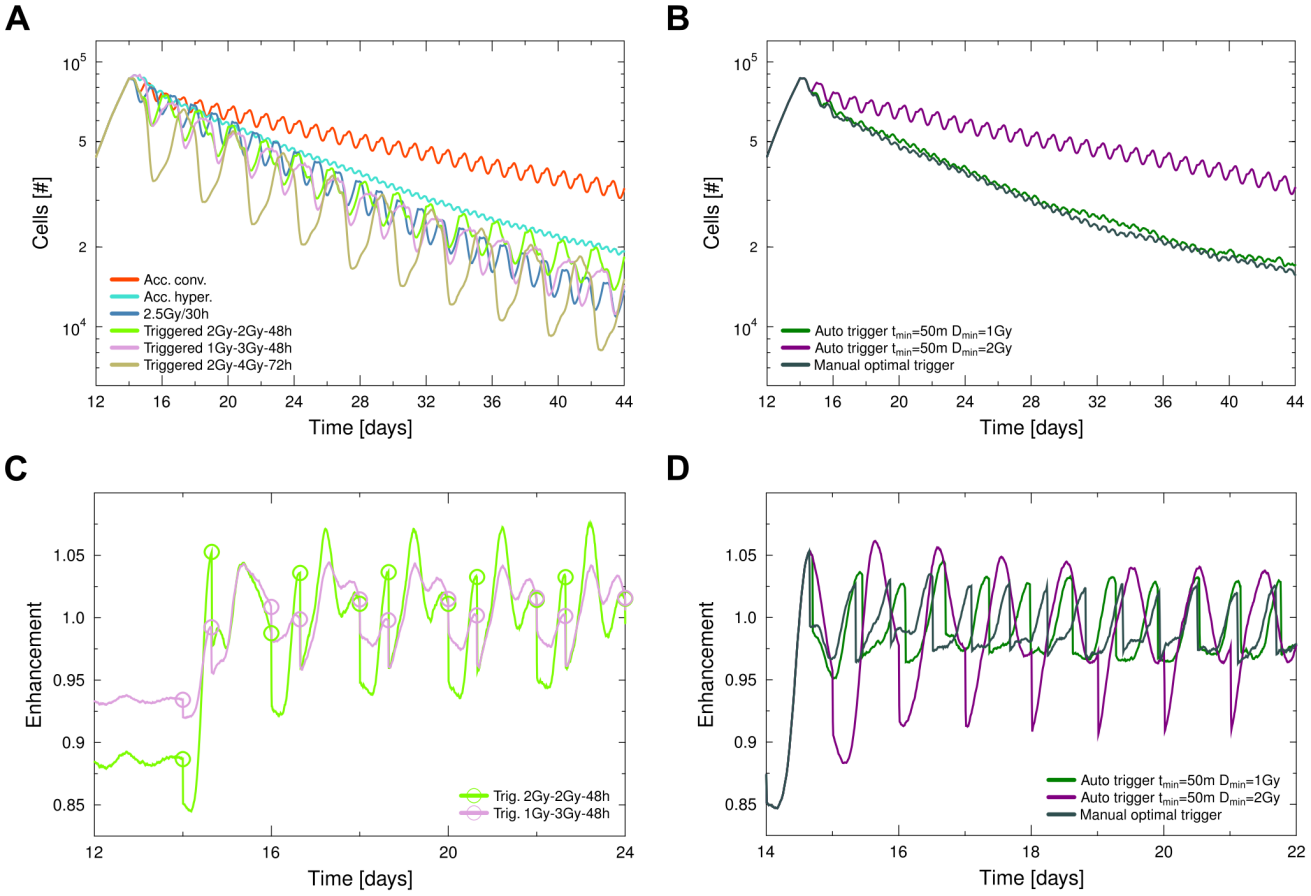
Performance of specific triggered irradiation schedules. Protocols were designed to induce a cell cycle response in the tumour which can be exploited in follow-up irradiations. **A** The overall reduction in tumour size achieved by different conventional and triggered schedules, which consist of trigger- and effector-dose followed by a pause to achieve a constant dose per time interval of 2 Gy/24 h. The performance of other triggered schedules can be found in the supplementary material figure S5. **B** Automatic triggering with a minimal inter-fraction time of 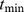 and a minimal dose of 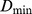 compared to full manual optimal triggering. **C** Example of a stable and unstable repeated sensitivity development in A. **D** Enhancement during automatic and full manual triggering in B (radiation timing marks omitted for visibility).

A range of tailored radiation protocols was designed in order to exploit the induced dynamic changes of radiosensitivity in the tumour and deliver radiation at timepoints of high enhancement (figure 6). One strategy was to divide the dose delivery into trigger-doses and subsequent effector-doses. Effector doses were delivered with a constant time-shift after the trigger-doses, which corresponded to the peak-timing in enhancement which was observed after administration of a single dose (figure 3). After each combined trigger and effector dose block, irradiation was paused in order to achieve an overall constant dose per time interval of 2 Gy/24 h.

In general, protocols were successful which used a smaller trigger dose in combination with a larger follow-up dose. The initial trigger dose induced a synchronisation in the tumour and increased enhancement. The large following effector dose would then be delivered to a sensitive tumour. Very small trigger doses below 1 Gy induced only a partial resynchronisation of the population and thus lead to an overall poor performance when employed in triggered schedules.

Surprisingly the protocol which delivers a trigger dose of 2 Gy followed by an effector dose of 4 Gy was able to cancel out the high regrowth which resulted from the pause of 48 h in between an effector dose and the next trigger-effector combination. Except for the fact that this protocol employs large single doses of 4 Gy (which might increase side-effects), it is especially interesting for a combination with adjuvant approaches which could reduce regrowth during the treatment pauses and thus could further improve the outcome substantially.

All triggered treatment protocols resulted in an increase in tumour reduction when compared to the standard accelerated conventional or accelerated hyperfractionated schedule. However, the simple altered protocol of constant 2.5 Gy/30 h was still the most successful protocol in terms of overall tumour burden reduction (figure 6). In this case the timing of the follow-up dose by chance persistently matched the peak in triggered sensitivity over the whole treatment time (figure 5).

In contrast, while the initial trigger-effector dose combination achieved the desired effect of inducing and exploiting a state of high radiosensitivity, the trigger-effector block of the same timing would not always prove to be right at later times during the irradiation regimen (figure 6). In many cases a fixed timing for the trigger-effector block would lead to the delivery of the effector dose at times of lowered radiosensitivity, once the tumour composition had changed during treatment. The time for the tumour to settle into a steady state in terms of enhancement reaction was larger than 48 hours and therefore larger than the typical inter-fraction time. Constant schedules which included longer pauses thus were able to maintain a proper trigger-effector dose timing for a part of the treatment regimen before changes in the tumour composition caused the timing to fail.

In many cases after application of the effector dose, a further strong peak in enhancement developed (figure 6). In principle, this allows for an increasing “stacking” of trigger and effector doses up to the case of continuous delivery at the next triggered sensitivity peak. Protocols with a combination of 3 consecutive well-timed doses in a constant block however did not prove to be effective, as delivery suffered strongly from the shift of the enhancement response during treatment.

As the enhancement response timing changes during the course of a prolonged treatment regimen, the targeting of the optimal enhancement point is only possible with permanent recalculation of the timing and, thus, can usually not be achieved with a fixed schedule. In order to exploit the build-up of radiosensitivity, triggering algorithms were tested which automatically delivered a follow-up dose at times of high enhancement (figure 6). A peak in enhancement was detected either by linear regression of the enhancement in a time window of interest, or in the simplest case by absence of an increasing enhancement value within a time window of 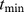. Once a peak was detected, radiation was delivered if the resulting dose was above a minimum of 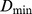. The dose was calculated in order to reach a constant dose per time interval of 2 Gy/24 h. For comparison a manually optimised schedule was tested, where a dose was always delivered exactly at the suitable enhancement peak.

The simple automatic triggering algorithm performed significantly better than conventional schedules, if the delivery of low doses was allowed by setting 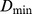 to 1 Gy. As a result of the small time interval which was necessary in order to identify each enhancement peak, the automatic triggering performs slightly worse than a manual optimised treatment schedule (figure 6).

While this automatic dose delivery could achieve a very good performance in terms of tumour reduction, it was still slightly inferior to the most successful schedule of 2.5 Gy/30 h. This inferior performance was due to the fact that the triggering algorithms and also manual scheduling performed only a *local* optimisation, triggering at the next suitable maximum of enhancement. However, an effective overall treatment schedule design requires a *global* optimisation, which cannot be achieved with algorithms that only take into account the following sensitivity maximum.

## Discussion

We employed an agent-based model in order to study the reaction of a microtumour to radiotherapy with special emphasis on the cell cycle distribution, synchronicity changes and the subsequent development of the overall radiosensitivity. The two-peaked increase in radiosensitivity which followed a dose of irradiation (figure 3) was used as a guideline for optimal irradiation timing in fractionated treatment regimens. The simple use of experimentally determined cell cycle-specific radiosensitivity, combined with a simple survival model, thus predicts optimisation possibilities in radiation delivery. The predicted results must must be validated or refuted in either an *in vitro* or an *in vivo* system.

The total possible gain or loss in efficiency of a treatment schedule due to cell cycle effects is immense. This becomes evident when the best and worst possible outcome for irradiation with 2 Gy are compared with according cell survival of 30% or 70%, depending on the cycle phase. For a treatment regimen with only 20 fractions this will yield a worst-case difference of a factor 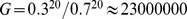. Even if this value represents an extreme case, most regimens will actually feature more than 20 fractions so that even small changes in survival based on cell cycle-dynamic can significantly alter the overall chances of tumour control.

In general the suppression of quiescent cells achieved by most hyperfractionated schedules is beneficial on one hand, as it will avoid quiescent radio-resistance. On the other hand, it will fully activate the growth potential of the tumour and thus allow for an exponential regrowth. The latter effect is especially devastating when combined with longer treatment pauses. An efficient combination with regrowth-cancelling adjuvant treatments would be needed, which could be combined with treatment protocols that make use of large inter-fraction pauses. Another viable option for combination of adjuvant chemotherapy and radiotherapy is the use of drugs which can prepare the tumour into a radiobiologically sensitive state [68], [69]. This can be achieved by the well-timed administration of drugs which have a cell-cycle synchronising effect, such as hydroxyurea [70], [71]. Within the simulation appropriate radio-chemo-schedules were tested and able to achieve significant enhancements in treatment outcome, especially when used in conjunction with high single doses (results not shown).

The observed cell cycle effects and reoxygenation of the tumour spheroid are also of interest for modern heavy-ion irradiation whenever spread out Bragg peaks are used that show a mixed-LET composition [72]. Especially in treatments which employ large single doses, such as in relativistic plateau proton-radiosurgery [73] or Stereotactic Body Radiation Therapy [66], [74], the cell cycle effects could be considerable and at the same time their dynamics can be easily estimated. Also in modern oligofractionated schedules which employ very high fractions [67], cell cycle effects could accordingly affect the treatment efficiency and could be possibly used quite actively. In order to study these effects *in silico* new radiation damage models need to be considered, which accurately describe radiation effects also in the range of very high doses [75]–[79].

While the exact timing of the effects will vary by cell- and radiation type, the universal effects such as the transient periods of radiosensitivity and radioresistance are present in every tumour and should subsequently be further studied within *in vitro* experiments. Variation of cell parameters such as quiescence radiation resistance, damage dynamics parameters, cell death durations and quiescence criterion led to minor *quantitative* changes, but the *qualitative* finding of transient radioresistant and radio-sensitive periods was conserved. The readiness of cells to enter and leave quiescence is of special interest, as it can increase the dampening of the oscillatory response in enhancement. Furthermore, the cell cycle duration and its typical variation are important for the sensitivity timing. Even for high variations of the typical cycle durations, which has been assumed in the simulation, the enhancement effects were pronounced and could be used for treatment optimisation. The specific nature of cell cycle checkpoint regulations (or the loss of it) and their genomic basis were disregarded in the present model. If a particular cell line is under consideration the status of key regulatory genes such as TP53 or ATM can be taken into consideration for refinement of the cell behaviour within the model [80].

The presented model rests on a foundation of very basic assumptions for the radiation reaction which are justified in most cells: first, cells exhibit a variation in radiosensitivity between different cell cycle phases [81], second, cells are subject to a degree of cell cycle regulation in response to damage or due to environmental effects (such as oxygenation, nutrient support or pressure) [38], [82], and third, cells in quiescence will show a resistance to radiation [83]. Ergo the described cell cycle effect should be present in any tumour system in which these assumptions are justified, irrespective of cell type or composition, although they may overlap or even be completely masked by other effects, e.g. reoxygenation dynamics.

Considering the overall development of radiosensitivity in a tumour which is triggered by irradiation, it seems reasonable to apply a scheme of trigger- and follow-up-doses to exploit the induced dynamics as was proposed and tested. Protocols which use a small trigger dose followed by a larger effector dose aimed at periods of high sensitivity could in principle be used clinically without alteration of the overall dose-rate. However, the identification of the transient periods of increased radiosensitivity is mandatory, as a wrong timing could result in a decrease of efficiency. When a multi-fractionated regimen is applied, the timing of irradiation cannot be simply derived from the sensitivity development in response to a single irradiation dose. Instead the development of sensitivity will be more complex, as the internal dynamics of the tumour (especially reactivation and depletion of quiescent cells) play an important role. With the use of simple automatic enhancement-based scheduling algorithms a significant increase in treatment performance was achieved. Triggering based on the monitoring of cell cycle-based enhancement is thus a possible method to automatically design optimised schedules. Such schedules would be robust as they can adapt to dynamic changes of the tumour and would furthermore be largely independent of any undetermined tumour parameters. In order to use any optimised scheduling approaches, the identification of high and low-enhancement periods is mandatory. Thus, live monitoring, or at least a higher sampling frequency combined with a model for the periods in between two measurements, is required to allow for a stable exploitation of the potential of cell cycle synchronisation effects.

While a higher frequency of monitoring induces additional clinical workload it is in principle simple to achieve, especially with combined PET/CT installations which are increasingly available at clinical treatment sites. A higher imaging frequency is also called for in conjunction with related phenomena such as hypoxia dynamics [84], where it has been shown that temporal variations of pO2 in mouse models exhibit 18-fold fluctuations with patterns on the scale of only minutes [85]. This observation clearly illustrates that measuring key tumour attributes only once or twice during a prolonged therapy regimen is not sufficient to understand or even therapeutically employ the kinetics of cell cycle redistribution or reoxygenation.

An experimentally or even clinically accessible observable for the synchronisation of the cell population is thus of utmost importance and should be the target of future investigations. If the orderedness of the cell cycle distribution can be assessed, its correlation with the radiosensitivity enhancement could be used to predict optimal irradiation times (see supporting figure S1). Another approach could be the monitoring of oxygen or glucose uptake in the tumour with high temporal resolution, as is regularly called for in the context of hypoxia [84]. This uptake is related to the collective development of the cycle distribution and therefore the overall radiosensitivity. In the best case a continuous monitoring of vital parameters such as cell cycle durations, key gene expressions and so forth would be available by a combination of imaging and possibly also sequential biopsies in order to predict suitable irradiation intervals.

In summary this suggests a basic scheme for the inclusion of cell cycle effects in therapy. In a first step the degree of cell cycle redistribution in the tumour which occurs in response to a treatment should be assessed. This assessment can also take into account a known genetic profile for cycle regulation and deregulation in the tumour. If the tumour is found to be susceptible to cell cycle redistribution and regulation, a synchronisation-based fractionation scheme should be considered [71]. The prediction of radiation sensitivity timings can thus be achieved using a basis of simulations and monitoring or biopsies with cultures of primary tissue. In the ideal case a feedback between modelling and measuring can be achieved, where information from only a few biopsies will be combined with a model in order to predict suitable patient-specific irradiation timings.

## Supporting Information

Figure S1 **Correlated development of orderedness and enhancement during tumour growth and irradiation.** In response to irradiation with 4 Gy at day 14 enhancement is strongly correlated with orderedness. If the orderedness of the cell population can be assessed experimentally, it can be used for the prediction of radiosensitive time windows.
(TIF)

Figure S2
**Radiation survival used within the simulation.** Cell-cycle phase specific survival data for V79 Chinese hamster cells has been used as example for radiation survival in this simulation [2]. **A** Survival curves include the average survival of cells in S-phase, survival of radio-resistant quiescent cells (using an effective dose reduction by a factor of 1.5 which follows measurements by [47]), and the expected survival 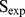 for the weighted cell cycle times from EMT6 cells used for calculation of the enhancement *E*. The spread in between survival of radioresistant S-phase cells and sensitive M-phase cells grows larger with increased dose, which is reflected in a higher possible variation of the enhancement *E* as illustrated in panel **B**.(TIF)

Figure S3 **Comparison of spheroid growth and histology** ***in silico*** **and** ***in vitro*** **.** Growth of EMT6/Ro cells as spheroids under different nutrient conditions was used to validate the model and is shown in panel **A** in comparison to experimental results from [43]. Panel **B** shows a thin central cutslice of a typical spheroid with an outer actively proliferating rim, an intermediate layer which is rich in quiescent cells and a hollow necrotic core partially consisting only of cell debris. Scale bar size in the figure is 100μm. Qualitative equality of the *in silico* and *in vitro* spheroids can be verified by comparison of the cutsection to experimental results such as the one presented in [92], figure 2.
(TIF)

Figure S4 **Visualisation of a tumour spheroid at different times during a hypofractionated schedule.** The spheroid was seeded at 0 h using 10 cells and grew undisturbed for 336 hours (upper row). Upon commencement of a high dose-per-fraction treatment of 4 Gy/24 h a destruction of the spheroid integrity through the dissolution of apoptotic cells was observed which led to the subsequent formation of smaller cell aggregates (middle row). In a stirred liquid medium the spheroid would accordingly dissolve. The last dose of the schedule is applied at 768 h after which cessation of treatment led to a fast regrowth of the tumour spheroid (bottom row).
(TIF)

Figure S5 **Triggered schedules and the development of enhancement.** **A** Radiation schedules which applied a small trigger dose in combination with a correctly timed effector dose were in general more successful in tumour burden reduction. The potential for synergy with an adjuvant chemotherapy is high, especially for triggered schedules which employ longer treatment pauses. **B** While a conventional 2 Gy/24 h schedule did not induce a persistent high enhancement in the tumour the 2.5 Gy/30 h schedule led to an increasing enhancement which was stable at a high level throughout the whole regimen.
(TIF)

Figure S6 **Timing of enhancement and dose delivery can explain the nonlinear dependency between inter-fraction time and number of fractions needed for sterilisation.** Enhancement details corresponding to the schedules shown in figure 5. While an interval of 1000 min still results in repeated delivery of the dose to a sensitive tumour a slightly increased interval will lead to delivery within resistant time windows. The associate change in total doses needed for sterilisation of the tumour is considerable as seen in figure 5.
(TIF)
