# Supplementary figures and images for: In Silico Analysis of Cell Cycle Synchronisation Effects in Radiotherapy of Tumour Spheroids
Source: PLoS Comput Biol. 2013 Nov 14;9(11):e1003295. doi: 10.1371/journal.pcbi.1003295 (PMC3828142; doi:10.1371/journal.pcbi.1003295)

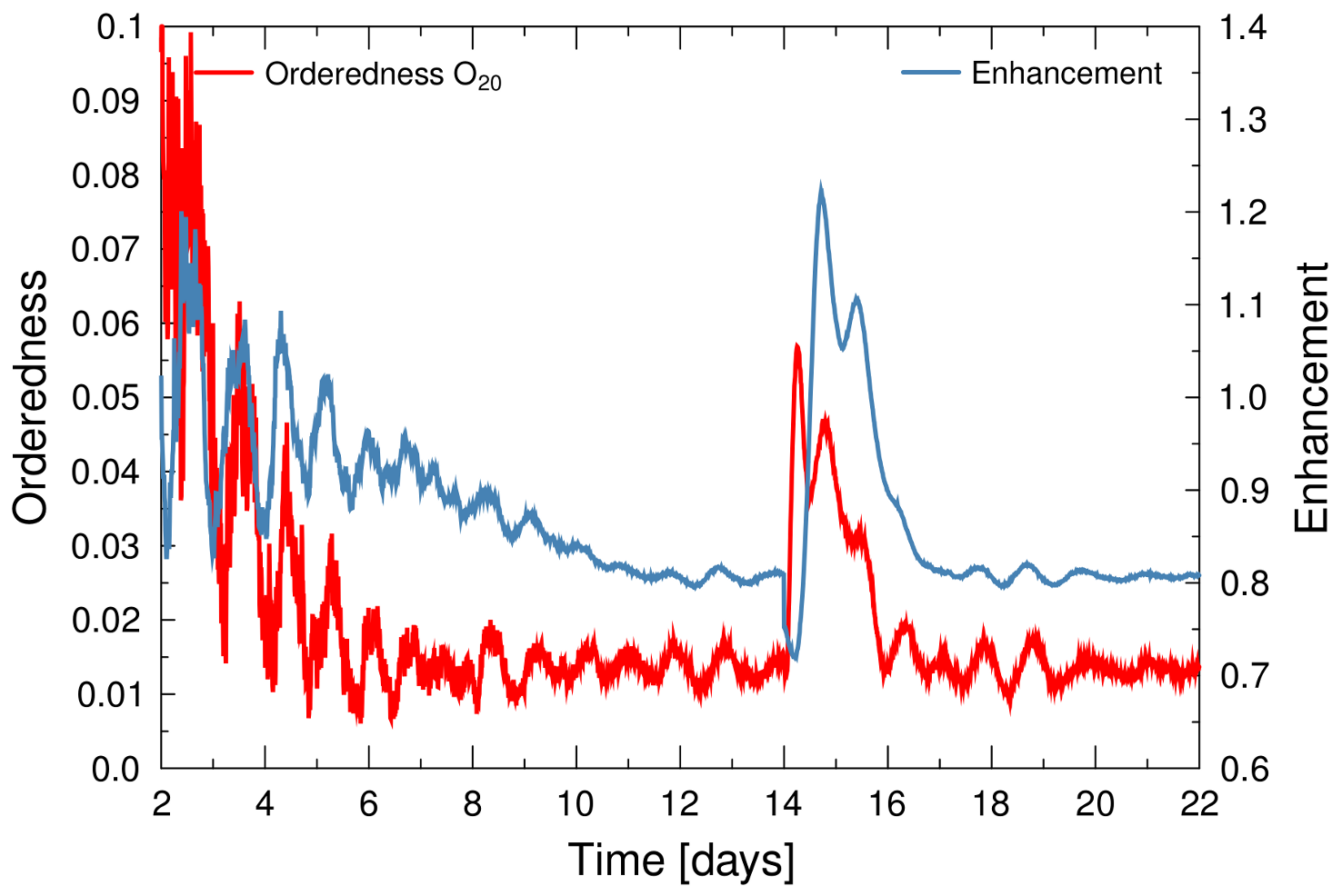

Supplement: Figure S1 — Correlated development of orderedness and enhancement during tumour growth and irradiation. In response to irradiation with 4 Gy at day 14 enhancement is strongly correlated with orderedness. If the orderedness of the cell population can be assessed experimentally, it can be used for the prediction of radiosensitive time windows. (TIF) [file pcbi.1003295.s001.tif]

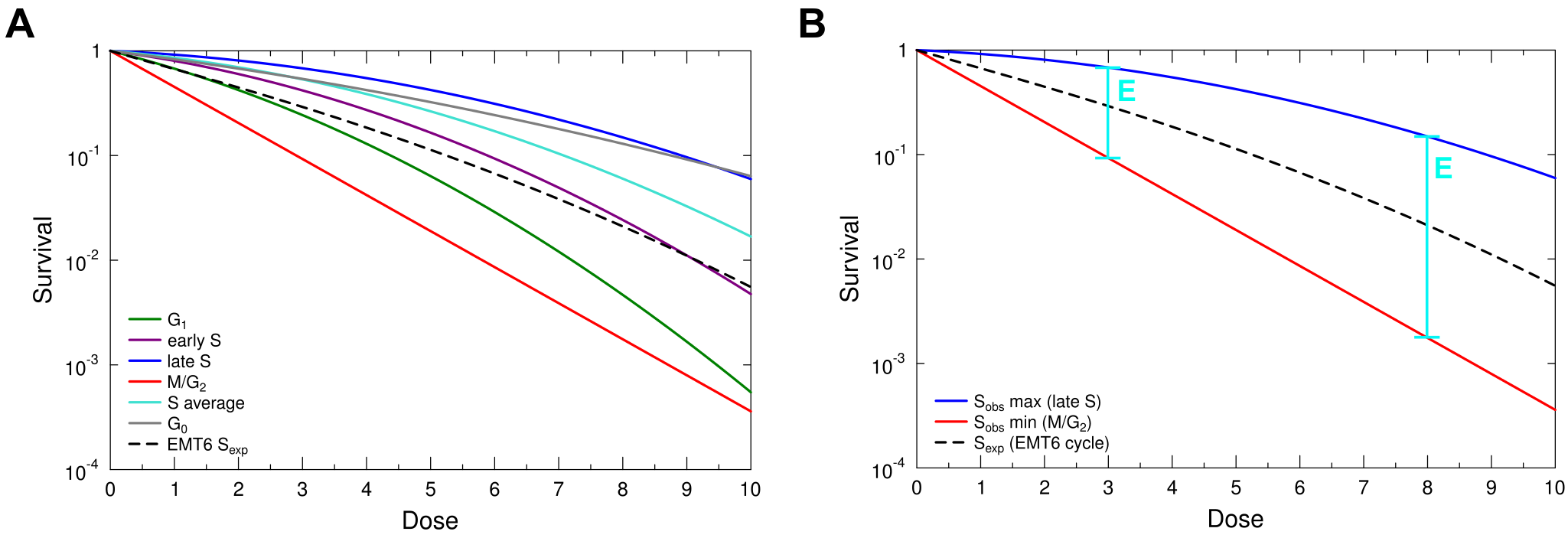

Supplement: Figure S2 — Radiation survival used within the simulation. Cell-cycle phase specific survival data for V79 Chinese hamster cells has been used as example for radiation survival in this simulation [2]. A Survival curves include the average survival of cells in S-phase, survival of radio-resistant quiescent cells (using an effective dose reduction by a factor of 1.5 which follows measurements by [47]), and the expected survival for the weighted cell cycle times from EMT6 cells used for calculation of the enhancement E. The spread in between survival of radioresistant S-phase cells and sensitive M-phase cells grows larger with increased dose, which is reflected in a higher possible variation of the enhancement E as illustrated in panel B. (TIF) [file pcbi.1003295.s002.tif]

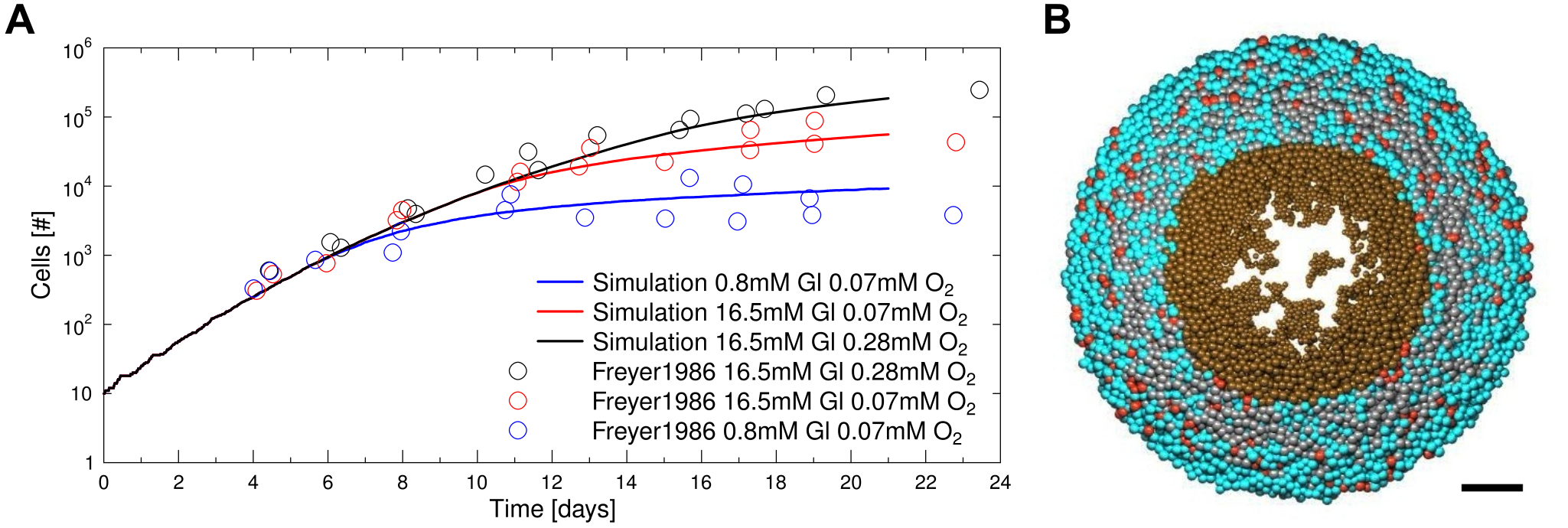

Supplement: Figure S3 — Comparison of spheroid growth and histology in silico and in vitro . Growth of EMT6/Ro cells as spheroids under different nutrient conditions was used to validate the model and is shown in panel A in comparison to experimental results from [43]. Panel B shows a thin central cutslice of a typical spheroid with an outer actively proliferating rim, an intermediate layer which is rich in quiescent cells and a hollow necrotic core partially consisting only of cell debris. Scale bar size in the figure is 100μm. Qualitative equality of the in silico and in vitro spheroids can be verified by comparison of the cutsection to experimental results such as the one presented in [92], figure 2. (TIF) [file pcbi.1003295.s003.tif]

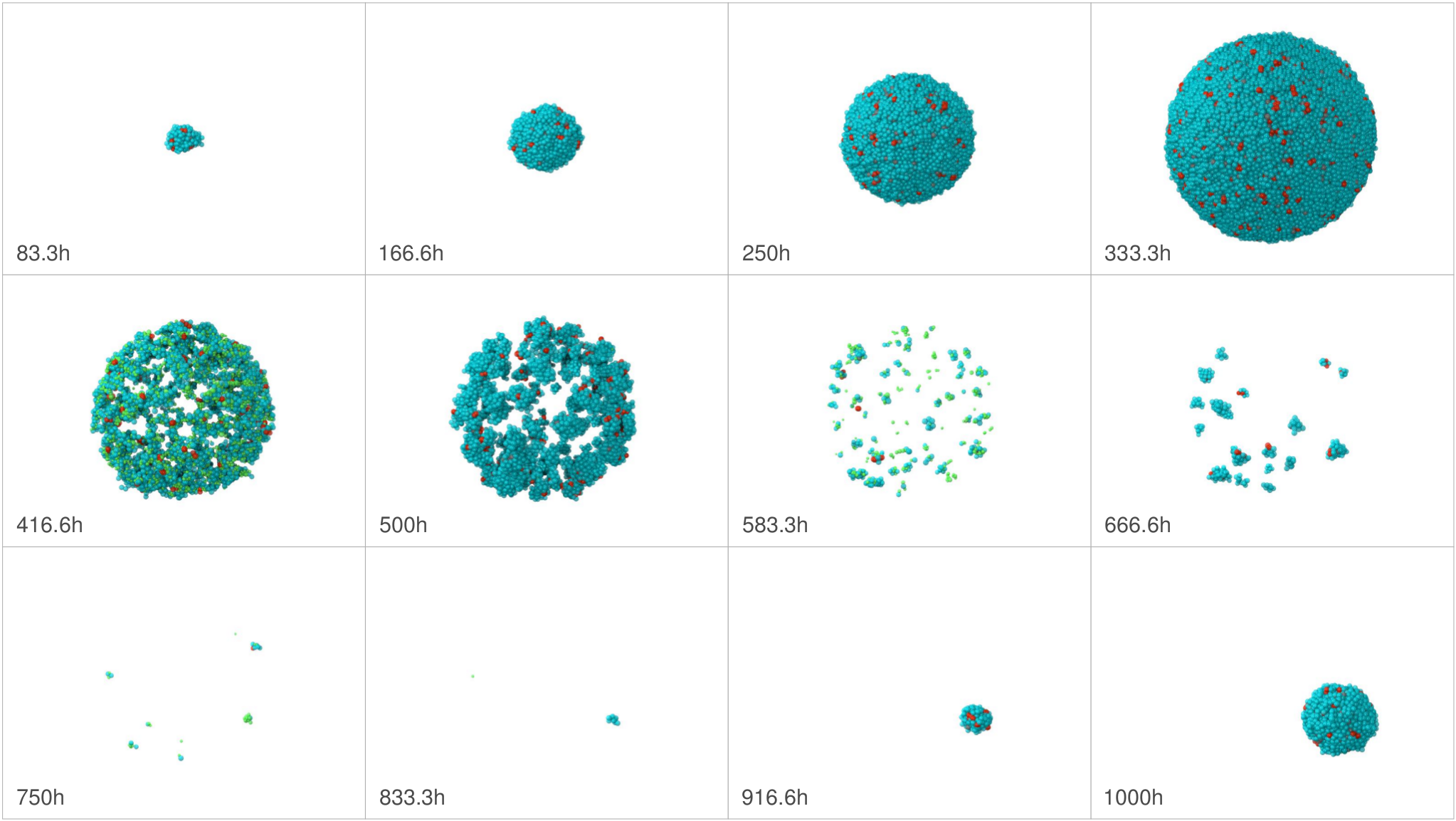

Supplement: Figure S4 — Visualisation of a tumour spheroid at different times during a hypofractionated schedule. The spheroid was seeded at 0 h using 10 cells and grew undisturbed for 336 hours (upper row). Upon commencement of a high dose-per-fraction treatment of 4 Gy/24 h a destruction of the spheroid integrity through the dissolution of apoptotic cells was observed which led to the subsequent formation of smaller cell aggregates (middle row). In a stirred liquid medium the spheroid would accordingly dissolve. The last dose of the schedule is applied at 768 h after which cessation of treatment led to a fast regrowth of the tumour spheroid (bottom row). (TIF) [file pcbi.1003295.s004.tif]

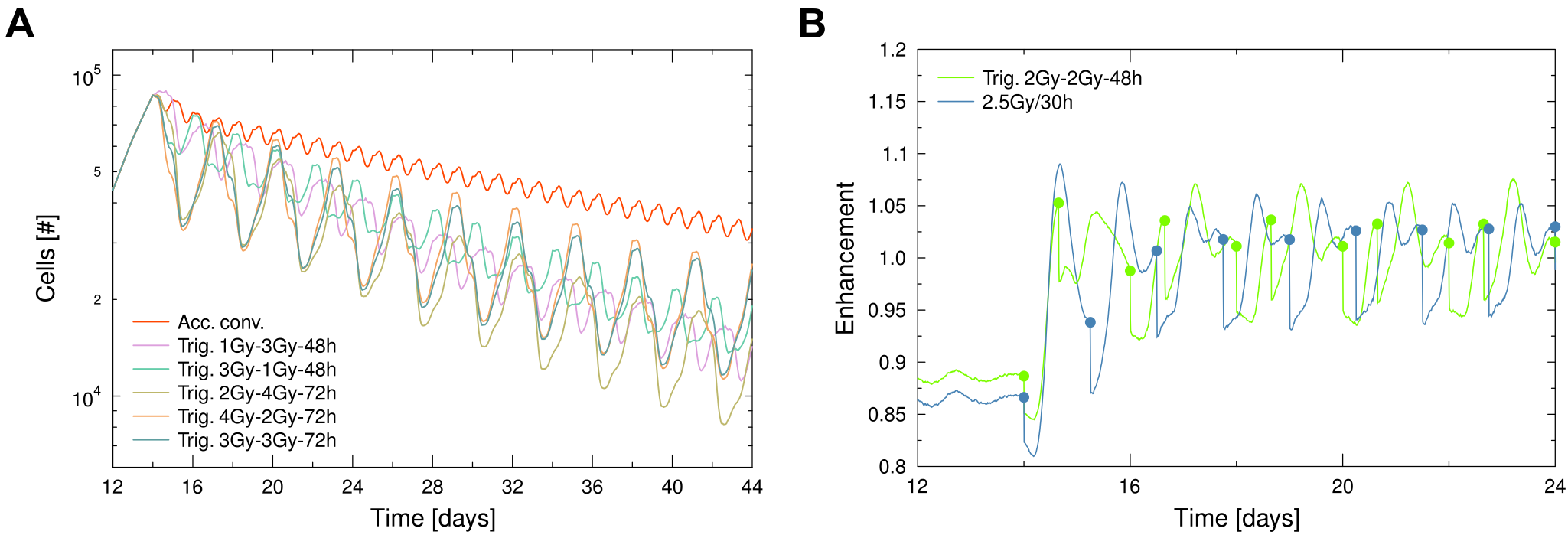

Supplement: Figure S5 — Triggered schedules and the development of enhancement. A Radiation schedules which applied a small trigger dose in combination with a correctly timed effector dose were in general more successful in tumour burden reduction. The potential for synergy with an adjuvant chemotherapy is high, especially for triggered schedules which employ longer treatment pauses. B While a conventional 2 Gy/24 h schedule did not induce a persistent high enhancement in the tumour the 2.5 Gy/30 h schedule led to an increasing enhancement which was stable at a high level throughout the whole regimen. (TIF) [file pcbi.1003295.s005.tif]

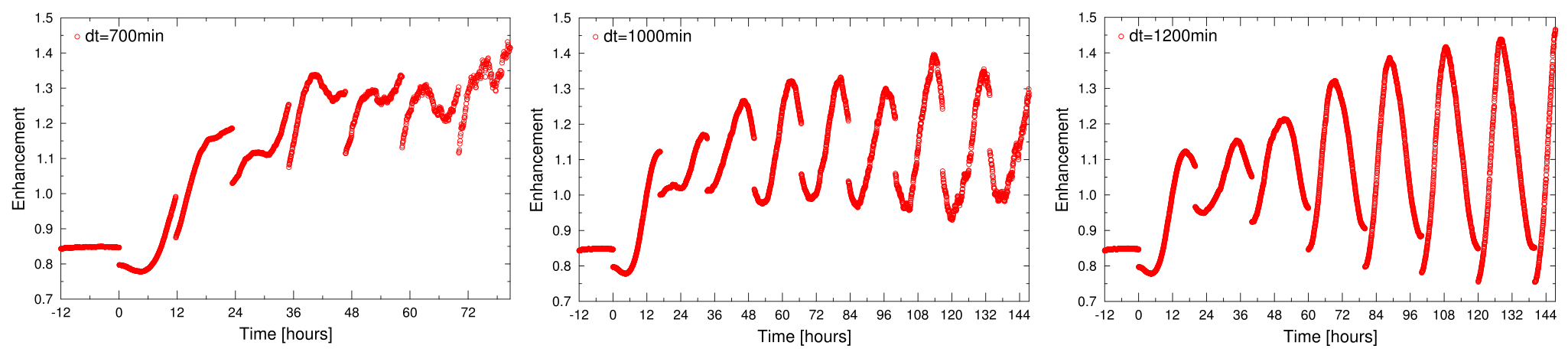

Supplement: Figure S6 — Timing of enhancement and dose delivery can explain the nonlinear dependency between inter-fraction time and number of fractions needed for sterilisation. Enhancement details corresponding to the schedules shown in figure 5. While an interval of 1000 min still results in repeated delivery of the dose to a sensitive tumour a slightly increased interval will lead to delivery within resistant time windows. The associate change in total doses needed for sterilisation of the tumour is considerable as seen in figure 5. (TIF) [file pcbi.1003295.s006.tif]
